# Epidemiology of HTLV-1 Infection and ATL in Japan: An Update

**DOI:** 10.3389/fmicb.2020.01124

**Published:** 2020-05-29

**Authors:** Masako Iwanaga

**Affiliations:** Department of Clinical Epidemiology, Graduate School of Biomedical Sciences, Nagasaki University, Nagasaki, Japan

**Keywords:** epidemiology, human T-cell leukemia virus type 1, HTLV-1, adult T-cell leukemia-lymphoma, ATL

## Abstract

Adult T-cell leukemia-lymphoma (ATL) is an aggressive T-cell malignancy caused by human T-cell leukemia virus type 1 (HTLV-1) infection that often occurs in HTLV-1-endemic areas, such as Japan, the Caribbean islands, Central and South America, Intertropical Africa, and the Middle East. In Japan, the nationwide estimation of the number of HTLV-1 carriers was at least 1.08 million in 2006–2007. Furthermore, in 2016, the nationwide annual incidence of newly infected with HTLV-1 was first estimated to be 3.8 per 100,000 person-years based on the age-specific seroconversion rates of blood donors in almost all areas of Japan. The incidence rate was three times higher in women than in men, and it was estimated that at least 4,000 new HTLV-1 infections occur yearly among adolescents and adults in Japan. As well known that HTLV-1 infection alone is not a sufficient condition for ATL to develop. To date, a variety of molecular abnormalities and host susceptibilities have been reported as candidate progression factors for the development of ATL in HTLV-1-carriers. In particular, quite recently in Japan, a variety of immunosuppressive conditions have been recognized as the most important host susceptibilities associated with the development of ATL from HTLV-1-carrier status. Furthermore, in 2013–2016 in Japan, a new nationwide epidemiological study of ATL was conducted targeting patients newly diagnosed with ATL in 2010–2011, from which the most current knowledge about the epidemiological characteristics of Japanese patients with ATL was updated as follows: (1) continuing regional unevenness of the distribution of people with HTLV-1, (2) further aging, with the mean age at diagnosis being 67.5 years, (3) declining M/F ratio, (4) increase of the lymphoma subtype, (5) sex differences in subtype distribution, (6) age differences in subtype distribution, and (7) comorbidity condition. In particular, 32.2% of ATL patients had comorbid malignancies other than ATL. However, the number of deaths due to ATL in Japan has been relatively stable, at around 1,000 patients annually, without significant decline from 1999 to 2017. Because the current epidemiological evidence about HTLV-1 and ATL is insufficient, further epidemiological studies are required.

## Introduction

Adult T-cell leukemia-lymphoma (ATL) is a T-cell malignancy caused by human T-cell leukemia virus type 1 (HTLV-1). ATL was first discovered as a distinct clinical entity (a mature, peripheral T-cell malignancy) in Japan in 1977 (Takatsuki et al., 1977; Uchiyama et al., 1977), followed by the discovery of the causative agent, HTLV-1 (Poiesz et al., 1980; Yoshida et al., 1982). The establishment of the etiological association between HTLV-1 infection and ATL was based on the following epidemiological and clinical facts: (1) all patients with ATL have antibodies against HTLV-1 (Hinuma et al., 1981, 1982), (2) geographical areas of high incidence of patients with ATL closely correspond with areas of high incidence of HTLV-1 carriers (The T-, and B-Cell Malignancy Study Group, 1985), (3) HTLV-1 immortalizes human CD4 T cells *in vitro* (Hattori et al., 1981), and (4) all ATL cells have monoclonal integration of HTLV-1 proviral DNA (Yoshida et al., 1984).

In the current World Health Organization classification of tumors of hematopoietic and lymphoid tissues (Oshima et al., 2017), ATL is defined as “a mature peripheral T-cell neoplasm composed of highly pleomorphic lymphoid cells and is caused by HTLV-1.” However, ATL cells involve not only hematopoietic/lymphoid tissues, but also a wide variety of human tissues including skin, spleen, lung, liver, CNS, and cardiac valve (O’Mahony et al., 2008; Oshima et al., 2017; Abolbashari et al., 2018). Typical ATL cells express CD2, CD3, CD4, and CD5 but usually do not express CD7 or CD8 (Oshima et al., 2017). Furthermore, ATL cells frequently express two specific markers of natural T regulatory cells: CC chemokine receptor 4 (CCR4) (Yoshie et al., 2002) and Forkhead box P3 (FoxP3) (Karube et al., 2004).

The diagnostic criteria for ATL and the classification of four clinical subtypes (acute, lymphoma, chronic, and smoldering subtypes) were proposed for the first time by the Japanese Lymphoma Study Group in the early 1990s (Shimoyama, 1991) based on the prognosis of patients enrolled in several nationwide hospital-based surveys in 1983–1987 (Tajima, 1990). Although several problems prevent diagnosing ATL based on the Shimoyama’s subtype classification, those criteria and the four clinical subtypes have still been useful for treatment decisions and are used widely in the current clinical setting.

The majority of individuals with HTLV-1 infection and patients with ATL have been reported in Japan, the Caribbean islands, Central and South America, Central and South Africa, Aboriginal regions in Central Australia, parts of the Middle East and Melanesia, parts of Europe, and other small regions (IARC, 1996; Proietti et al., 2005; Gessain and Cassar, 2012). Moreover, even within such endemic areas, further clustering of people with HTLV-1 infection and patients with ATL have been recognized, particularly in Japan (Satake et al., 2012, 2015; Sagara et al., 2018).

The earlier version of this review summarized the literature published up to 2012 in Japan (Iwanaga et al., 2012). This review includes additional information published after 2012, focusing on the epidemiological aspects of people with HTLV-1 infection and patients with ATL in Japan. However, there have still only been a few prospective cohort studies intended to reliably assess the incidence rates of ATL among asymptomatic people with HTLV-1 infection. Instead, a variety of study settings, such as nationwide surveys and regional population-based studies, were published. Thus, readers should keep in mind that each epidemiological study has its own limitations in terms of case accumulation and population setting.

## HTLV-1 Seroprevalence

Since the discovery of HTLV-1 in Japan in the early 1980s, the overall nationwide number of people with HTLV-1 infection has been estimated several times by multiplying the sex- and age-specific seroprevalence of HTLV-1 screening test results among first-time blood donors by age- and sex-specific demographic data across the whole of Japan in a specific year.

The first estimation of the age-specific HTLV-1 prevalence in Japan was done in 1983 by using HTLV-1 screening test results from approximately 3,000 blood donors aged 40–64 years in selected areas in Japan (Maeda et al., 1984) and identified that HTLV-1 seroprevalence was higher in Southwest Japan and in elderly individuals. Later, Hashimoto et al. (1991) investigated the nationwide age-specific prevalence of HTLV-1 by including approximately 610,000 blood donations from the entire area of Japan, confirming that the HTLV-1 seroprevalence was higher in female than male individuals and that the prevalence was higher in older people (Table 1).

**TABLE 1:** HTLV-1 seroprevalence among Japanese blood donors in literature.

**References**	**Maeda et al., 1984**	**Hashimoto et al., 1991**	**Satake et al., 2012**
Year of screening	1983	1988	2006–2007
Areas screened	64 Blood centers	77 Blood centers	All 47 prefectures
Screening test method	IF	PA, IF	PA, IF
No. screened	12,800	614,879	1,196,321
No. HTLV-1-positive	241	11,586	3,787
Overall seropositive rate (%)	8.0	1.88	0.32
Seropositive rate (%) by age group	(Only Kyusyu area)	M/F	M / F
16–19 years	2.0	2.3/2.9	0.12 / 0.11
20–29 years	2.6	4.4/4.8	0.17 / 0.20
30–39 years	3.8	8.0/10.1	0.22 / 0.27
40–49 years	5.1	10.3/21.0	0.57 / 0.63
50–59 years	6.2	13.7/14.3	0.97 / 1.26
60–64 years			1.29 / 1.66
70–79 years	N.D.	N.D.	1.59 / 2.36
80–89 years	N.D.	N.D.	1.92 / 2.96
90–99 years	N.D.	N.D.	2.19 / 3.48

*HTLV-1 = human T-cell leukemia virus type 1; PA = particle agglutination; IF = immunofluorescence, N.D. = not done.*

**FIGURE 1:**
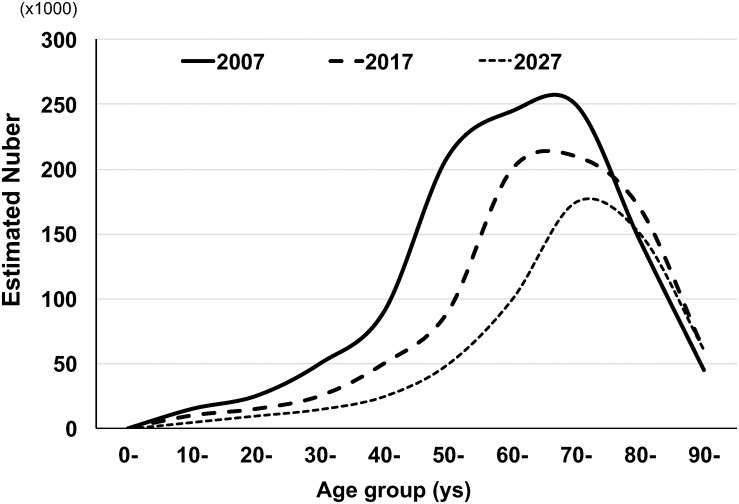
Possible changes of estimated numbers of HTLV-1 carriers in Japan by age group. The figure was modified from Figures 3 and 5 in the article by Satake et al. (2012). The age-specific estimated numbers of HTLV-1 carriers in 2007 were determined by multiplying the age-specific HTLV-1 positive rate of blood donors in 2007 by the age-specific general population. The age-specific estimated numbers of HTLV-1 carriers in 2017 were also obtained by Satake et al. Based on the change in the age-specific estimated numbers of HTLV-1 carriers between 2007 and 2017, Satake et al. estimated the future (2027) age-distribution of age-specific HTLV-1 carriers.

The most recent age-specific HTLV-1 prevalence and estimated number of HTLV-1-infected people in whole Japan were reported by Satake et al. (2012) based on the HTLV-1 screening test results from approximately 1,200,000 blood donations during 2006–2007 throughout all of Japan. The results showed that 3,787 were confirmed to be positive for HTLV-1 antibodies. The positive rate was from 0.12 to 1.59% for male donors and from 0.11 to 2.36% for female donors in age brackets ranging from 16–19 to 90–99 years (Table 1). Based on those age-specific HTLV-1 seroprevalence rates during 2006–2007, Satake et al. estimated that the number of HTLV-1 carriers was at least 1.08 million in Japan in 2006–2007. In addition, based on the fact that the highest age-specific HTLV-1 seroprevalence rates shifted toward older age groups from 1983 to 2006–2007, Satake et al. estimated that the number of HTLV-1 carriers will decrease by half in the next two decades in Japan (Figure 1).

## Incidence of New HTLV-1-Infection

Although knowledge about the pattern and trends of HTLV-1 seroprevalence is important for public health, information about the incidence of new HTLV infection is also important and essential for planning prevention efforts (Table 2).

**TABLE 2:** Summary of incidence of HTLV-1 infection in Japan in literature.

**References**	**Publication year**	**Study design**	**Study population**	**HTLV-1 seroprevalence**	**No. population at risk**	**No. incidence persons**	**Incidence rate estimate**
Stuver et al., 1993	1993	Prospective cohort	Married couples in the Miyazaki Cohort	Men :23.5% Women : 31.2%	Men : 761 Women: 1,063	7	1.2 per 100 PYs among seronegative husbands with seropositive wives 4.9 per 100 PYs among seronegative wives with seropositive husbands
Takezaki et al., 1995	1995	Cross-sectional	Residents in an area endemic for HTLV-1	Age > 40 years: 27.0% Age < 40 years: 7.9%	999 in 1980 722 in 1990	Not available	3.3 per 1,000 PYs for men 6.7 per 1,000 PYs for women
Satake et al., 2016	2016	Retrospective cohort	Repeated blood donors in whole Japan	Varied by area from 0.13 to 1.07%	Men : 2100925 Women: 1274906	532	3.8 per 100,000 PYs for all 6.9 per 100,000 PYs for men 2.3 per 100,000 PYs for women

*Abbreviations: HTLV-1 = human T-cell leukemia virus type 1; PYs = person years.*

After the discovery of milk-bone transmission of HTLV-1 in Japan in the early 1980s, there were also several reports of heterosexual transmission of HTLV-1 in Japanese couples. Tajima et al. (1982) reported that the seroprevalence in wives with seropositive husbands (68%) was greater than that in wives with seronegative husbands (20%) and for the first time suggested the possible sexual transmission of HTLV-1. Later, Stuver et al. (1993) reported that the HTLV-1 seroconversion rate was 4.9 per 100 person-years among seronegative wives with seropositive husbands but 1.2 per 100 person-years among seronegative husbands with seropositive wives in a prospective cohort study of 534 older married couples in Miyazaki Prefecture, Kyushu district, where HTLV-1 is endemic. Takezaki et al. (1995) also reported that the HTLV-1 seroconversion rate was 3.3 per 1,000 person-years for men and 6.7 per 1,000 person-years for women in a village where HTLV-1 is highly endemic. All three of these reports were very informative, and the results were consistent in that seroconversion rates were higher in women than men. However, these previous studies were small, and no nationwide information on new HTLV-1 infection has been available. Therefore, in 2016, our Japanese research team first investigated the nationwide annual incidence of HTLV-1 infection based on the age-specific seroconversion rates of Japanese blood donors across Japan (Satake et al., 2016). That nationwide study estimated that the incidence of HTLV-1 in Japanese blood donors was 3.8 per 100,000 person-years. The incidence rate was three times higher in women than men (6.9 vs. 2.3 per 100,000 person-years; *p* < 0.0001). The incidence was also higher in earlier birth cohorts, probably because of differences in lengths of the sexual transmission. After extrapolating the incidence rate to the Japanese general population, it was estimated that at least 4,000 new HTLV-1 infections will occur yearly among adolescents and adults in Japan.

In Japan, several government measures and actions have been implemented in place to prevent new HTLV-1 infection, including HTLV-1 antibody screening for pregnant women, the recommendation for mothers with positive results to refrain from breastfeeding, and HTLV-1 antibody screening of all donated blood since 1986. However, there have been no strategies to prevent new HTLV-1 transmission among adolescents and adults. After publication of Satake’s report, HTLV-1 infection is now recognized as a sexually transmitted infection (STI) in Japan. Other measures to control new HTLV-1 infection by treating HTLV-1 as a type of STI are expected in Japan. Quite recently, the Japanese Ministry of Health, Labour and Welfare has started to act to decrease HTLV-1 infection in Japan (Nishijima et al., 2019). Further, HTLV-1 infection is now recognized as an international infectious agent, and it should not be ignored in medical training curriculum (Cook and Taylor, 2020).

## ATL Incidence

Most published studies have estimated the incidence of ATL in a group by simply merging the number of cases of ATL with the number of people in a population, such as demographic statistics, blood donors positive for HTLV-1, or an existing group of HTLV-1 carriers. However, few prospective studies have investigated the incidence of ATL in asymptomatic people with HTLV-1 infection.

### Global Incidence of ATL

**FIGURE 2:**
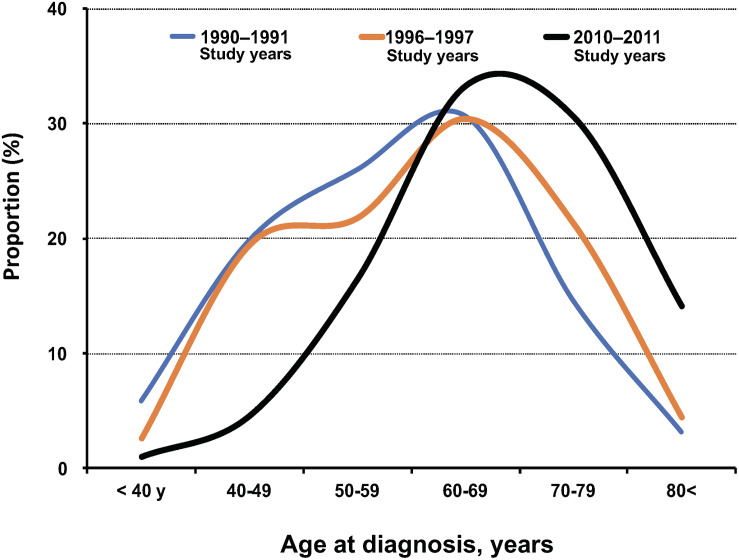
Summary of age at diagnosis of ATL by study year (1990–92, 1996–97, and 2010–11) in Japan in literature. The data in each diagnosis period were cited from the literature.

The International Agency for Research on Cancer (IARC) has regularly reported the global burden of infection-associated cancers, such as *Helicobacter pylori* for gastric cancer, hepatitis B virus and hepatitis C virus for liver cancer, human papillomavirus for cervical cancer, Epstein–Barr virus for lymphoma, human herpes virus type 8 for Kaposi’s sarcoma, and HTLV-1 for ATL. According to the GLOBOCAN database of the IARC, the worldwide estimated number of newly diagnosed ATL cases in the years 2002, 2008, and 2012 were 3,340 (Parkin, 2006), 2,100 (de Martel et al., 2012), and 3,000 (Plummer et al., 2016), respectively (Table 3). All the reports showed that patients with ATL were predominantly male and older than 50 years. Furthermore, in the IARC 2008 report (de Martel et al., 2012), the total number of 2,100 ATL patients consisted of 1,500 patients in more developed regions (71.4%) and 660 patients in less developed regions (28.6%) (Table 3). The data of the higher burden of newly diagnosed patients with ATL in more developed regions than less developed ones were extremely rare among infection-associated cancers: in most of which new cases were usually more frequent in less developed than more developed regions. The burden of newly diagnosed patients with ATL in more developed regions is apparently influenced by the fact that Japan is the country with the highest number of HTLV-1 and ATL cases in the world. The updated worldwide estimated number of newly diagnosed ATL cases was reported to be 3,000 in 2012 (Table 3). That number is similar to that of 3,340 patients in 2002 but greater than the 2,100 new patients in 2008, and the difference was particularly great among people older than 50 years. The international burden of newly diagnosed patients with ATL in older age groups may also be influenced by the fact that the average age at ATL diagnosis has dramatically shifted toward older adults, according to the recent Japanese nationwide ATL study (Figure 2) (Nosaka et al., 2017). However, readers should keep in mind that the GLOBOCAN data are only based on well-organized cancer registry systems but information from other ATL endemic regions such as Africa are not taken into account because in Africa, the majority of cancer registries are still underdeveloped.

**TABLE 3:** ATL incidence in GLOBOCAN.

**Year of** **diagnosis**	**No. new** **cases**	**No.** **attributable to infection**	**Proportion of new cases attributable** **to each infectious agent (%) infectious agent (%)**	**No. attributableto infection,** **by age group**			**No. attributableto infection,** **by development status**	
				**<50 years**	**50–69 years**	**≥70 years**	**Less developed**	**More developed**
2006	3,340	3,340	100%	NA	NA	NA	550	2,790
2008	2,100	2,100	100%	580	980	580	660	1,500
2012	3,000	3,000	100%	630	1200	1200	–	–

### ATL Incidence in Japan

In Japan, several epidemiological studies have been conducted to estimate the annual incidence of ATL among HTLV-1 carriers and the general population, but the exact annual incidence of ATL in the general population has remained unclear. This was primarily because nationwide information about ATL incidence has not been reported in the Japanese cancer registry system until recently, except for a few cancer registry areas with high-quality data. One reason for this incredible situation was that ATL was included in the category of “*other leukemia*” in Japan because of its name of adult T-cell “leukemia.” However, after the establishment of the New Cancer Registry Law in Japan in 2013, the renovated Japanese cancer registry system started to treat ATL as an independent malignancy. The analyzed data of the new nationwide cancer registry system will be published in the near future. The latest annual incidence of ATL will be approximately 1,500 patients.

Although nationwide information about ATL is not currently available in the Japanese cancer registry system, several large-scale epidemiological studies have been conducted in Japan. Chihara et al. (2012) reported ATL incidence in the selected 15 population-based cancer registries with high-quality data in Japan, by dividing them into 12 “HTLV-1 non-endemic areas” and three “HTLV-1 endemic areas.” After analyzing 1,380 patients in the non-endemic areas and 2,055 patients in the endemic areas who were diagnosed in 1993–2006, they reported that the age-standardized incidence rate of ATL showed an increasing trend in the HTLV-1 non-endemic areas but was stable in the HTLV-1-endemic areas. The report suggested that the presence of ATL and HTLV-1 carriers has spread to areas that are non-endemic for HTLV-1. This fact suggests a need for preventive strategies against HTLV-1 transmission in Japan.

Apart from population-based studies, a large-scale pathological study conducted in the Kyushu-Okinawa district, where HTLV-1 infection is endemic, reported that ATL accounted for 51–59% of non-Hodgkin lymphoma (NHL) in 2000 (Arisawa et al., 2000). This proportion is much higher than that of a nationwide pathological study of NHL, which reported that ATL accounts for only 7.5% of all lymphomas (Lymphoma Study Group of Japanese Pathologists, 2000). The most recent large-scale pathological report mentioned that ATL accounted for 9.2–16.8% of NHL in the Kyushu-Okinawa district in 2016 (Miyoshi and Ohshima, 2018), indicating that ATL is still the most common type of malignant lymphoma across the entire area of Japan, although the proportion was lower than that reported in 2000 (Arisawa et al., 2000).

### ATL Incidence From Asymptomatic HTLV-1 Carriers and the Risk Factors

From early 1980s to early 2000s in Japan, several epidemiological studies estimated that the annual incidence rate of ATL from HTLV-1 carriers among general population in HTLV-1 endemic areas, and reported that the rate was approximately from 60 to 130 per 100,000 HTLV-1 carriers with male predominant (Tokudome et al., 1989; Tajima, 1990; Arisawa et al., 2000). Furthermore, previously, the lifetime risk of ATL among HTLV-1 carriers was estimated to be 4–6% for men and 2.6% for women in several HTLV-1-endemic areas (Kondo et al., 1989; Tokudome et al., 1989; Arisawa et al., 2000). Unfortunately, however, there has been no other studies to update information on the lifetime risk of ATL among HTLV-1 carriers in general Japanese population.

It has been well recognized that HTLV-1-infection alone is not sufficient to develop ATL from HTLV-1-carrier status. Regarding risk factors for the development of ATL from HTLV-1-carriers, so many risk factors have been documented in literature such as aging (Iwanaga et al., 2012), family history of ATL or HAM/TSP (Iwanaga et al., 2012), immunocompromised status, and a variety of genetic alterations that would related to HTLV-1 oncogenesis (Kogure and Kataoka, 2017). In a nationwide prospective study of HTLV-1 carries in Japan (named JSPFAD) reported that a higher proviral load (more than 4 copies/100 PBMCs) is an independent risk factor for progression of ATL, even after adjusting for sex, age, family history of ATL, and other possible risk factors (Iwanaga et al., 2010). The updated analysis in the JSPFAD study is underway.

### ATL Incidence From HTLV-1 Positive Patients With Specific Conditions

Recently, several clinical genetic/epigenetic studies of asymptomatic HTLV-1 carriers and patients with ATL have been conducted to identify the molecular entity responsible for the development and the prognosis of ATL (Yamagishi et al., 2012; Kataoka et al., 2015, 2018). Some of the recently identified molecular abnormalities are now considered as new therapeutic targets of ATL. However, the definite molecular entity that promotes the progression from HTLV-1 carrier status to the development of ATL has still not been conclusively identified. As with other cancers, the accumulation of several genetic/epigenetic abnormalities and host conditions together probably affects the development of ATL. Apart from genetic/epigenetic abnormalities, host susceptibility and several inflammatory conditions are known to be associated with the development of ATL from HTLV-1 carrier status.

#### Chronic Parasitic Infections

HTLV-1 carriers with abnormal immune systems are well-known to be at high risk of developing ATL. Several previous studies reported that HTLV-1 carriers co-infected with *Strongyloides stercoralis* are a high-risk group for developing ATL because of the clonal proliferation of HTLV-1-infected lymphocytes and high proviral load (Nakada et al., 1987; Yamaguchi et al., 1987; Plumelle et al., 1997; Gabet et al., 2000). Satoh et al. (2002) also suggested that *S. stercoralis* infection induces polyclonal expansion of HTLV-1-infected cells by activating the interleukin 2/interleukin 2 receptor (IL-2/IL-2R) system in dually infected carriers, which may be a precipitating factor for ATL. Furthermore, Nishi et al. (2016) reported that *S. stercoralis infecti*on was related to higher numbers of ATL-related deaths.

#### Immunosuppressive Status, Including Organ Transplantation

Patients with rheumatoid arthritis (RA), Sjögren syndrome, and organ transplantation use strong immune suppressive therapies for long durations. Such strongly immunosuppressive states are also considered as high risk for the development of ATL from such patients with HTLV-1. There have been a non-negligible number of clinical reports of ATL developing among HTLV-1-positive patients in immunosuppressive status (Eguchi et al., 1992; Tsurumi et al., 1992; Suzuki et al., 2006; Umekita and Okayama, 2020).

Furthermore, recently, a small but not negligible number of cases of the development of ATL from HTLV-1-positive recipients undergoing strong immunosuppressive treatment after living-donor renal and liver transplantation have been reported from not only Japan but also other countries (Kawano et al., 2006, 2018; Yoshizumi et al., 2012, 2016; Martin et al., 2014; Cook et al., 2016; Motomura et al., 2019; Yamauchi et al., 2019). One of these, Yoshizumi et al. (2012) reported five patients with transplantation-related ATL after intervals of 181–1,315 days among 82 living donor liver-transplant recipients who were asymptomatic HTLV-1 carriers prior to transplantation. Further, there have been four patients with transplantation-related HAM/TSP among 10 HTLV-1-negative recipients after transplants from HTLV-1-positive donors (Yamauchi et al., 2019). These reports together suggest that a strongly immunosuppressive state is a definite risk factor for the development of not only ATL but also HAM/TSP among asymptomatic HTLV-1 carriers.

#### Other Specific Conditions

Definite risk factors for the development of ATL from HTLV-1-carrier status have been well documented in many studies and I have also summarized regarding risk factors in the previous review article (Iwanaga et al., 2012). As for host susceptibility to develop ATL other than described above, early age at the time of HTLV-1 infection (vertical transmission), senior aging of HTLV-1-carrier (average age at diagnosis of ATL was 60 years old in Japan or 40 years in Jamaican and Brazilian), male sex is well described in many literature. As for laboratory markers, a high level of soluble interleukin-2 receptor more than 500 U/ml, and a higher HTLV-1 proviral load level more than 4 copies per 100 peripheral blood mononuclear cells are also well recognized in many literature. Regarding persons who infected with HTLV-1 via transfusion of HTLV-1-positive blood, there are many reports of the development of HAM/TSP (Osame et al., 1990), but so far there are no documented cases of ATL after transfusion of HTLV-1-positive blood.

## Update on Epidemiological Feature of ATL in Japan

The earlier version of this review summarized the literature published up to 2012 (Iwanaga et al., 2012). At that time, available nationwide epidemiological information about ATL for the whole of Japan was limited to the nationwide hospital-based surveys during 1983–1987 (Tajima, 1990) and the abridged version of the hospital-based survey in 2006–2007 (Yamada et al., 2011). Since then, information about epidemiological and clinical characteristics of patients with ATL has not been updated for the whole of Japan. However, a large-scale, multicenter, hospital-based survey was conducted in Japan in 2013–2016, targeting patients with ATL cases that were newly diagnosed in 2010–2011 (Nosaka et al., 2017). The study accumulated 996 patients with ATL diagnosed at 126 participating hospitals from all over Japan and identified the following seven epidemiological features of most current ATL in Japan (Tables 4, 5).

**TABLE 4:** Characteristics of patients with ATL during three different periods: 1984–1985, 1992–1993, and 2010–2011.

**Year of diagnosis of patients**	**1984–1985**	**1992–1993**	**2010–2011**
Total no. of patients	181	712	996
Evaluable no. of patients	NA	NA	922
M/F ratio	1.4	1.13	1.12
**Subtype**			
Acute	102(56.4%)	489(69.4%)	456(49.5%)
Lymphoma	38(21.0%)	151(21.4%)	237(25.7%)
Chronic	25(13.8%)	36(5.1%)	131(14.2%)
Smoldering	16(8.8%)	29(4.1%)	98(10.6%)
**Age at diagnosis, year**			
min, max (mean)	24,90(56.9)	25,87(58.9)	34,100(67/5)
**Age at diagnosis category**			
<40 years	NA	31 (4.4)	9 (1.0)
40–49	NA	133 (18.7)	42 (4.6)
50–59	NA	209 (29.4)	152 (16.5)
60–69	NA	191 (26.9)	307 (33.3)
70–79	NA	122 (17.2)	282 (30.6)
80<	NA	24 (3.4)	130 (14.1)
**Geographic area at diagnosis**			
Hokkaido/Tohoku	42(23.6%)	51(7.2%)	50(5.4%)
Kanto	9(5.1%)	35(4.9%)	59(6.4%)
Chubu-Hokuriku	1(0.6%)	91(12.8%)	55(6.0%)
Kinki	6(3.4%)	90(12.7%)	88(9.5%)
Chugoku-Shikoku	24(13.5%)	39(5.5%)	48(5.2%)
Kyushu-Okinawa	96(53.9%)	401(56.5%)	622(67.5%)

*ATL = adult T-cell leukemia-lymphoma; IQR = interquartile range; SD = standard deviation; NR = not reported; NA = figure was available but information on the number was not; SD = standard deviation.*

**TABLE 5:** Comorbidities of patients with ATL at diagnosis by subtype in a nationwide epidemiological study in Japan, 2010–2011.*

	**Total**	**ATL subtype**			
		**Acute**	**Lymphoma**	**Chronic**	**Smoldering**
**Total patients, *N***	922	456	237	131	98
**Past medical history**					
Transfusion before 1986, yes, *N* (%)	15(1.7)	NA	NA	NA	NA
Skin diseases, yes, *N* (%)	43 (4.8)	NA	NA	NA	NA
Infectious diseases, yes, *N* (%)	98 (10.9)	NA	NA	NA	NA
Malignancies, *N* (%)	108 (12.0)	NA	NA	NA	NA
Autoimmune diseases, *N* (%)	36 (4.0)	NA	NA	NA	NA
**Comorbidities at diagnosis**					
Any of diseases, yes, *N* (% of each subtype)	297 (32.2)	145 (32.2)	67 (28.5)	43 (33.6)	42 (43.8)
Hematological malignancies, yes, *N* (% of each subtype)	8 (0.9)	4 (0.9)	2 (0.8)	0 (0)	2 (2.0)
**Non-hematological malignancies, yes, *N* (% of each subtype)**	**113 (12.3)**	**53 (11.6)**	**21 (8.9)**	**17 (13.0)**	**22 (22.4)**
Infectious diseases, yes, *N* (% of each subtype)	93 (10.1)	53 (11.6)	16 (6.8)	14 (10.7)	10 (10.2)
Neurologic diseases, yes, *N* (% of each subtype)	21 (2.3)	13 (2.9)	4 (1.7)	3 (2.3)	1 (1.0)
Autoimmune diseases, yes, *N* (% of each subtype)	9 (1.0)	3 (0.7)	1 (0.4)	3 (2.3)	2 (2.0)

*This information was modified from data in Nosaka et al. (2017). ATL = adult T-cell leukemia-lymphoma; IQR = interquartile range; SD = standard deviation; NA = not available.

(1) *Sustained regional ubiquity:* Of the 922 evaluable patients with ATL diagnosed in 2010–2011, 67.5% were still diagnosed in the Kyushu–Okinawa areas, where HTLV-1 infection is most prevalent, followed by 9.5% in Kinki and 6.4% in Kanto, which are the most populated areas of Japan and have the largest population inflow from other areas, including Kyushu–Okinawa.
(2) *Further aging:* The mean age at diagnosis of ATL was 67.5 years in 2010–2011, which was older than the corresponding figures of 58.9 years in 1992–1993 and 56.9 years in 1984–1985 (Figure 2). The main reason for the current shift toward a more advanced age at the onset or diagnosis of ATL might be due to the amazing aging shift of the Japanese population.
(3) *Declining M/F ratio*: The male/female ratio of numbers of ATL patients in the 1980s was 1.4; therefore, at that time, ATL was considered as a male-predominant disease. However, after the 1990s, the male-female ratio declined toward 1. Nevertheless, by considering the male/female ratio of Japan’s general population in 2010, which was 0.95 [Portal Site of Official Statistics of Japan (e-Stat), 2019], ATL is still considered as a male-predominant disease in Japan. Detailed data of sex- and age-specific ATL incidence from the renovated Japanese cancer registry will be published in the near future.
(4) *Increase of lymphoma subtype:* Among all registered patients, the proportion of lymphoma subtype increased from 21.0% in 1984–1985 to 21.4% in 1992–1993 and 25.7% in 2010–2011 (Table 4).
(5) *Difference in subtype distribution by sex:* There was a significant sex difference in the distribution of subtypes: the frequency of chronic type was higher in female compared with male patients; conversely, other types were more frequent in male than female patients. In particular, the lymphoma type was more frequent in male patients (60%).
(6) *Difference in subtype distribution by age:* There was a significant age difference at diagnosis by subtype. Patients with chronic subtypes tended to be diagnosed at a younger age, whereas those with lymphoma subtypes were diagnosed at an older age.
(7) *Comorbid malignancies other than ATL*: Of all the patients with ATL diagnosed in 2010–2011, 32.2% had one or more comorbid diseases, including (in descending frequency order) hematological malignancies, non-hematological malignancies, infectious diseases (cytomegalovirus infection was the most common), neurologic diseases, autoimmune diseases, and other diseases (Table 5). Among comorbid non-hematological malignancies, colorectal cancer was the most common. This finding differed from that of a previous report by Arisawa et al. (2006), in which liver cancer was the most common among Japanese atomic bomb survivors with HTLV-1 infection.

For HIV-infected patients, although the development of AIDS-defining malignancies has been well known, the development of non-AIDS-defining malignancies becomes another growing problem, recently (Monforte et al., 2008). By considering that patients with ATL or HTLV-1 are also in an immunosuppressive state similar to that of HIV-infected patients, I personally think that it may be time to consider about concepts such as “non-ATL HTLV-1-defining cancer” or “non-ATL HTLV-1-defining malignancies” for HTLV-1 carriers and ATL patients, although a recent systematic review and meta-analysis denied an association between HTLV-1 infection and the development of any cancers other than ATL (Schierhout et al., 2020).

A possible reason for no significant association between HTLV-1 infection and any cancers other than ATL in the report of Schierhout et al. is that it might have been suffered to several limitations such that a half of evaluated data were all causes mortality for any cancers but not taken into account incidence rate of each cancer. Therefore, any cancers with a high cure rate are not reflected to the results. Furthermore, in their report, information on viral marker status such as HTLV-1 proviral load is lacked; therefore, it has been uncertain that the adverse health effect was definitely due to HTLV-1-infection.

## ATL Mortality

**FIGURE 3:**
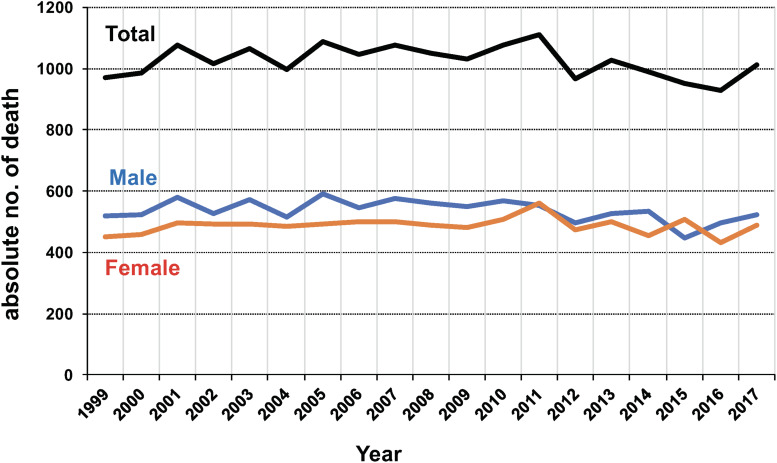
Annual numbers of deaths from adult T-cell leukemia (ATL) in Japan during 2001–2010. The data were obtained from vital statistics on the Portal Site of Official Statistics of Japan (e-Stat) (accessed March–April 2019). Abbreviations: M, male, F, female.

Unfortunately, information about the global burden of deaths from ATL is not available from any data source. Instead, information about deaths from ATL in Japan has been made available from “e-Stat” [Portal Site of Official Statistics of Japan (e-Stat), 2019] at regular intervals. As shown in Figure 3, the number of deaths due to ATL in Japan has remained at almost 1,000 patients every year without significant decline. The M/F ratio has remained almost stable, ranging from 0.88 to 1.20 throughout the period from 1999 to 2017.

## Concluding Remarks

Although many studies of epidemiological evidence about ATL and HTLV-1 carriers from Japan have been published, reliable information about the annual incidence of ATL in the setting of longitudinal prospective studies has still been limited. Existing published data regarding predisposing factors and genetic abnormalities are still insufficient to explain the characteristics of ATL oncogenesis. Additional unknown factors may be involved in the development of ATL from asymptomatic HTLV-1 carrier status. Further well-designed epidemiological studies are needed to fully understand the relationship between HTLV-1 infection and ATL.

In summary, in Japan, at least 1.1 million individuals are still infected with HTLV-1 (Satake et al., 2012), at least 4,000 individuals are newly infected with HTLV-1 (Satake et al., 2016), approximately 1,500 patients are newly diagnosed with ATL, and approximately 1,000 deaths from ATL are reported every year. For patients with ATL in Japan, several novel promising agents, such as mogamulizumab (humanized anti-CCR4 monoclonal antibody) (Ishida et al., 2015), and lenalidomide (Ogura et al., 2016) are currently being adopted. A phase III study of watchful waiting versus a combination of interferon alpha and azidothymidine (Bazarbachi et al., 2010) for symptomatic indolent ATL is now underway in Japan. Furthermore, other promising agents targeting zeste homolog 1/2 dependent epigenetic abnormalities (Yamagishi et al., 2019) and histone deacetylase (Zhang et al., 2019) in ATL cells are also expected to improve outcomes. Nevertheless, preventing new HTLV-1 infections and reducing the new incidence of ATL are still the major public health concerns in HTLV-1 endemic countries.

## Author Contributions

MI designed and wrote the manuscript.

## Conflict of Interest

The authors declare that the research was conducted in the absence of any commercial or financial relationships that could be construed as a potential conflict of interest.
